# Gambling policy positions of Finnish newspapers between 2004 and 2020: An automated content analysis

**DOI:** 10.1177/14550725221083438

**Published:** 2022-08-11

**Authors:** Jani Selin, Riku Nyrhinen

**Affiliations:** 3837 Finnish Institute for Health and Welfare, Helsinki, Finland; 3837 Finnish Institute for Health and Welfare, Helsinki, Finland

**Keywords:** automated content analysis, gambling, newspapers, policy, Wordfish

## Abstract

**Aims:** The media can influence gambling policy formation and public opinion. Previous research has established that the tension between political or public interest in gambling revenue and gambling harm is fundamental for understanding gambling policy. There are two opposing gambling policy positions: (1) gambling revenue or the economic benefits of gambling, and (2) the harmful impacts of gambling. This study is the first study to estimate these gambling policy positions of newspapers on a common scale. The objective is to estimate how the gambling policy positions of major Finnish daily newspapers evolved between 2004 and 2020. This knowledge deepens our understanding about the changes in the relative balance between harm and revenue in gambling policy. **Methods and data:** The data consisted of newspaper editorials (*N* = 58) on gambling policy from five major Finnish daily newspapers between 2004 and 2020. The data were analysed with the automated content analysis algorithm Wordfish. **Results:** The results show that there has been a clear shift in the gambling policy positions of the major Finnish newspapers towards increased acknowledgement of the importance of prevention and reduction of gambling harm. **Conclusions:** Due to the interplay between the media, politics, and the public, it is likely that the importance of prevention and reduction of gambling harm will be recognised and addressed to a larger extent when gambling policy is formulated in Finland in the future. More generally, if the gambling policy positions of media and other stakeholders change, this can facilitate a promotion of harm prevention policies.

Even in the current age of social media, traditional media still plays an important role in influencing public opinion and political agenda (Klinger & Svensson, 2015; Langer & Gruber, 2020; Luo et al., 2019; Van Aelst & Walgrave, 2011). While the exact extent of the direct influence of media on politics is open to debate (Van Aelst & Walgrave, 2011), there is evidence of an interplay between the media and politics and politicians (Klinger & Svensson, 2015; Vliegenthart et al., 2016). The media and other actors such as different interest groups can influence the political agenda; that is, what the issues are that matter and need to be addressed (Birkland, 2007; Van Aelst & Walgrave, 2011; Vliegenthart et al., 2016). In democratic societies, media can have an important role in agenda-setting and governments often face situations “where they simply cannot ignore public sentiment without risking the loss of legitimacy or credibility, and must give the issue some priority on the agenda” (Jann & Wegrich, 2007, p. 47). Media influence on the political agenda can sometimes result in policy change (Fawzi, 2018; Langer & Gruber, 2020; Van Aelst & Walgrave, 2011). However, media’s capacity to influence policy is dependent on many contextual factors: the relevance and quality of the news content, interests of the policy actors, and the relationships between policy actors (Yanovitzky & Weber, 2019). The media can also help politicians to bring new issues to the agenda or keep old issues on the agenda. The relationship between the media and politics can therefore be grasped as one characterised by reciprocity (Klinger & Svensson, 2015; Vliegenthart et al., 2016).

Newspaper editorials are one of the most direct ways newspaper media can influence public and political agendas (e.g., Liu & Hood, 2019). Editorials can be considered as “votes” in favour of or against a policy (see Ho & Quinn, 2008) or calls to address an issue. Moreover, editorials express, to adopt the widely used spatial metaphor in political science, the *policy position* of a newspaper on a specific issue or policy field, for example social policy (Ho & Quinn, 2008; Kaneko et al., 2021). An example of a newspaper’s policy position is the endorsement of a proposal for banning smoking in restaurants in the editorial of Finnish *Helsingin Sanomat* in 2005 (The Editorial Board, 2005).

There is a long research tradition in political science that focuses on estimating policy or ideological positions of political parties from political texts on a common scale (e.g., Koljonen et al., 2020; Lowe et al., 2011). This line of research on is typically focused on the spatial positions of the political parties on a left–right dimension or a liberal–conservative dimension, the assumption being that “a set of party positions, whether a cross-section or a time series, can be located on some (continuously defined) metric scale” (Lowe et al., 2011, p. 125). Policy positions related to specific policy dimensions such as foreign policy or social policy have also been studied (Lauderdale & Herzog, 2016; Lowe et al., 2011). The most used data source on the policy positions of different political actors, cross-sectionally and longitudinally, is text (Lowe et al., 2011). Policy positions are latent variables that cannot be directly observed from texts (Koljonen et al., 2020).

In the literature on addiction policies, the level of the alcohol and tobacco control policies in different countries have been measured on a metric scale by applying the basic ideas of the policy positions approach (e.g., Joossens & Raw, 2006; Karlsson & Österberg, 2001). The same approach has been used to estimate the level of national gambling control policies in the European Union member countries (Lindeman et al., 2015). The more policy interventions there are, the higher the level of control and the more health and social impacts of the product in question are taken into account in policy formation and implementation (Karlsson & Österberg, 2001). Conversely, the fewer the policy interventions, the more likely it is that instead of health and social impacts of the products, the financial interests of the relevant industries, governments and other stakeholders are foregrounded in the policy formation and implementation. The opposite ends of this policy dimension are thus economic benefits and harmful impacts of the products. In this article this dimension will be called the *revenue–harm dimension*. If the economic aspects of gambling are foregrounded when gambling policy is discussed, the revenue end of the dimension is emphasised. If the harmful impacts of gambling on individuals, groups or society are foregrounded, the harmful end of the dimension is emphasised. A key premise of this article, supported by extensive research evidence, is that the tension between political or public interest in gambling revenue and gambling harm is fundamental for understanding gambling policy space in Finland and globally (Adams, 2016; Adams & Livingstone, 2015; Marionneau & Nikkinen, 2020; Selin, 2019; Sulkunen et al., 2019; Wardle et al., 2019). One example of this tension is the way the fiscal interest of governments is likely to make effective harm prevention and reduction policies more difficult to adopt (Adams, 2016; Marionneau & Nikkinen, 2020; McMahon et al., 2019; Sulkunen et al., 2019).

Insofar as the media can influence political and public agenda, the policy position of media is something politicians consider. According to Törrönen (2004) the policy positions of the newspapers, as expressed in editorials, matter because the editorials are “responsive indicators of the opinion climate and of the shifts, fluctuations, and changes going on in the power relationships between societal actors and policies” (p. 62). Thus, knowledge of the policy positions of newspapers (or other key stakeholders) at different points in time deepens our understanding about the changes in the relative balance between harm and revenue in gambling policy. The knowledge on policy positions can open possibilities for follow-up studies, as well as for studies that seek to explain or predict changes in the balance between harm and revenue (Lauderdale & Herzog, 2016; Slapin & Proksch, 2008). To our knowledge, there is no previous research on the gambling policy positions of the media, the fourth estate. This study is thus the first attempt to fill this gap in gambling research by analysing the changes in the gambling policy positions of the Finnish mainstream newspapers with the help of a state-of-the-art automated content analysis method. The objective of the study is to estimate how the gambling policy positions of major Finnish newspapers evolved between 2004 and 2020.

The state-owned gambling monopoly Veikkaus is the only legal gambling operator in mainland Finland.^1^ Veikkaus was created as a result of merging the three previous state-controlled gambling monopolies in 2017. The justification of the legal framework and the main objective of gambling policy is prevention and reduction of gambling harm (Selin, 2019). Policies with an aim to prevent and reduce harm have also been introduced between 2004 and 2020, including, for example, restrictions on marketing, 18-year age limit, and mandatory player identification for electronic gambling machines (Karsimus & Günther, 2021; Selin, 2019). Gambling revenue is distributed to a wide array of beneficiaries, the state included. Over the past few years, the media, citizens, researchers, politicians and other stakeholders have increasingly started to criticise the marketing and other practices of Veikkaus, and in light of the apparent interest of the state and other stakeholders in gambling revenue, to question the official justification of the regulatory framework (e.g., Lakka, 2019; Rämö, 2018; YLE, 2020). Moreover, a previous study (Lerkkanen & Marionneau, 2019) shows an upward trend in the number of articles on gambling and gambling harm in the biggest Finnish daily newspaper, *Helsingin Sanomat*. Based on these facts, we hypothesise that between 2004 and 2020 there has been a shift in the policy positions of Finnish newspapers from emphasis of the importance of gambling revenue to paying increasing attention to gambling harm.

## Data and method

### Data

The raw data consisted of newspaper editorials (*N* = 58) on gambling policy from five major Finnish daily newspapers between 2004 and 2020. All the newspapers declare to be politically neutral. The newspapers had the highest number of readers in print and online (total reach) in 2020 (Kansallinen mediatutkimus, 2021). The newspapers were (total reach in brackets): *Helsingin Sanomat* (1,841,000), *Turun Sanomat* (379,000), *Aamulehti* (562,000), *Kaleva* (429,000) and *Keskisuomalainen* (263,000). Only the electronic archives of *Helsingin Sanomat*, *Turun Sanomat* and *Kaleva* covered the whole observation period (see Table 1, and Appendix A in the supplemental online material). Due to the COVID-19 pandemic, we only had access to the electronic archives of the newspapers, and it is possible that some editorials published by *Aamulehti* or *Keskisuomalainen* are missing from the raw data (Table 1). The editorials included in the data needed to fulfil the following criteria: no named author, published between 2004 and 2020, length at least 100 words, gambling policy as main subject matter. The year 2004 was chosen as the starting point because discussions over amendments to the Lotteries Act (1047/2001), which had come into effect in January 2002, commenced that year (Selin, 2019).^2^

**Table 1.** The numbers of editorials by year and by newspaper.

Year	*HS*	*TS*	*KV*	*AL*	*KSML*	Total
2004	1	0	0	n/a	n/a	1
2006	2	0	0	n/a	n/a	2
2007	2	2	0	n/a	n/a	4
2008	2	3	0	n/a	0	5
2009	1	0	0	n/a	0	1
2011	0	3	0	n/a	0	3
2012	2	0	0	n/a	0	2
2013	1	0	0	n/a	0	1
2014	1	1	0	n/a	0	2
2015	5	0	1	1	0	7
2016	2	0	0	2	0	4
2017	2	0	1	2	0	5
2019	4	3	5	2	3	17
2020	3	1	0	0	0	4
Total	28	13	7	7	3	58

*Note*. *HS* = *Helsingin Sanomat*; *TS* = *Turun Sanomat*; *KV* = *Kaleva*; *AL* = *Aamulehti*; *KSML* = *Keskisuomalainen*.

### Wordfish

Wordfish is an *unsupervised* statistical method or algorithm for the scaling or estimation of latent policy positions on the basis of textual data (Grimmer & Stewart, 2013; Slapin & Proksch, 2008). Unsupervised automated content analysis methods use “properties of the texts to estimate a set of categories and simultaneously assign documents … to those categories” (Grimmer & Stewart, 2013, p. 15). Supervised methods instead need texts that are usually categorised by human coders and on the basis of this categorisation the “algorithm then ‘learns’ how to sort the documents into categories” (Grimmer & Stewart, 2013, p. 9). For this study, Wordfish was the obvious choice because there is no established way to place the Finnish newspapers on the revenue–harm dimension. The use of hand coding of the editorials was not possible in the absence of an elaborated and validated coding scheme (Slapin & Proksch, 2008). An expert survey on all investigated editorials would have been possible, but we lacked the resources for such a time-consuming endeavour (Slapin & Proksch, 2008). The basic benefit of automated content analysis methods is in their ability to manage large data sets with less time and resources than approaches needing human coding (Grimmer & Stewart, 2013). Moreover, automated content analysis produces quantified estimates of policy positions on a metric scale, making it easier to conduct longitudinal and comparative studies (Grimmer & Stewart, 2013).

Wordfish estimates policy positions on a single policy dimension solely based on the observed *word frequencies* in text documents (Grimmer & Stewart, 2013; Slapin & Proksch, 2008). Wordfish assumes that word frequencies follow a Poisson distribution, the rate of which is affected by four parameters: a set of actor-fixed effects (α), a set of word-fixed effects (ψ), a set of word-specific estimates for the importance of a word in discriminating between policy positions (β), and a set of estimates of actors’ policy position on a one-dimensional scale (ω). The model follows a reparametrisation of Goodman’s (1979) row–column association model, where the count of the *j*th word in the *i*th document, y_ijt_, is a Poisson process with a rate conditional on the document's position, ω_i_, with an added time-series estimation variable, *t*. In this study, the time variable was combined into *i* so that every step represents both a new document and a new point in time:

$$y_{ijt}∼Poisson(\lambda_{ijt})$$

$$\log\;\lambda_{ijt}=\alpha_{it}+\psi_{j}+\omega_{it}\beta_{j}$$

The algorithm uses an iterative form of expectation maximisation (EM) based on a Poisson regression model, a type of linear regression, modelling the relationship between known-dependent and unknown-independent variables through maximum likelihood estimates. The iteration alters between an expectation (E) step, which creates a function for the expectation of the log-likelihood using the current iteration's estimate for a pair of word/actor parameters, and a maximisation (M) step, which maximises the log-likelihood conditional on the expectation by using the Broyden–Fletcher–Goldfarb–Shanno algorithm. The EM implementation consists of five steps and alternates between word and actor parameter pairs as the latent variables. The process is repeated until an acceptable level of convergence is reached (for details see online supplementary Appendix B; Slapin & Proksch, 2008).

Confidence intervals for actor positions and word weights can be estimated using the information matrix of the likelihood conditional on the word parameter estimates, by using a parametric bootstrap solution as explained by Slapin and Proksch (2008). In this study, the solution offered by Slapin and Proksch was used in the way it was implemented in Wordfish 1.3 for R language. First, the script estimates all parameters by running the expectation maximisation algorithm described above. The estimates are used to calculate word frequencies for each cell in the corresponding word-document matrix. The script then generates *N* new matrices, filling them with random draws from a Poisson distribution with parameter lambda for each cell in the word-count matrix. In this study, the default value for *N* was set at 500. After the matrices have been populated, the script reruns the EM algorithm on each of these simulated matrices – using the original maximum-likelihood estimates as starting values – and estimates 500 new policy positions. The script then approximates the 95% confidence interval by using 0.025 and 0.975 quantiles of the simulated policy positions.

Bunea and Ibenskas (2017) mention critical issues that need to be considered when applying Wordfish. First, the policy dimension needs to be well defined on the basis of prior knowledge. We consider that the revenue–harm dimension under investigation here is well defined (see discussion above). Second, the texts analysed are assumed to contain information relevant to the policy dimension. When the data were collected, only editorials that discussed gambling policy were included. Editorials discussing other aspects of gambling were excluded.^3^ Third, Wordfish also requires that “texts are similar (and thus comparable) in terms of their authorship, text generation process, targeted audience and communication purpose” (Bunea & Ibenskas, 2017, p. 343). Editorials as data fulfil these conditions. In addition, the length of the texts and number of unique words in the texts are important for the validity of the policy position estimates (Proksch & Slapin, 2009; Slapin & Proksch, 2008). In this study the relatively low annual number of gambling policy editorials and the fact that editorials are typically quite short texts was a challenge, but the consequences of this were mitigated by pooling editorials into larger groups (for details, see discussion below). Wordfish also assumes that “the probability that each word occurs in a text is independent of the position of other words in the text” (Slapin & Proksch, 2008, p. 708). While it is obvious that this assumption is not how we choose words when writing or talking, the model produces results that are sufficiently valid (Grimmer & Stewart, 2013).

### Validation of the policy position estimates

The policy estimates produced by an unsupervised method require the use of different validation techniques. In this study, predictive validity was used to test the results (Grimmer & Stewart, 2013). In the absence of any previous estimates of the policy positions of the newspapers we cross-validated our Wordfish estimates by comparing them to the results of an expert survey, a standard practice in literature (Slapin & Proksch, 2008). We asked two groups (*N* = 18) of researchers and other experts familiar with the revenue–harm dimension to estimate the gambling policy positions of the editorials of *Helsingin Sanomat* (*N* = 13) from 2014, 2015, 2019 and 2020 on a scale from one to five, where one indicated that the focus of the text was on the economic aspects of gambling policy. These years were chosen because the preliminary Wordfish estimates indicated that in 2014 and 2015 the policy positions were among the closest to the revenue end of the policy dimension and in 2019 and 2020 they were among the closest to the harm end of the dimension. A group of ten experts estimated the positions of six editorials and a group of eight experts estimated the positions of seven editorials. Substantive validation (Grimmer & Stewart, 2013) of the results was conducted by careful reading of the words with highest and lowest weights. This strategy was used to verify that the policy dimension captured by Wordfish corresponds meaningfully to the revenue–harm dimension.

### Data pre-processing

The Wordfish manual (Proksch & Slapin, 2009) recommends the removal of irrelevant information from the texts. To determine what is irrelevant, it is necessary to clearly define and represent the different structural elements that appear in texts. Thus, in natural language processing tasks one of the goals of text pre-processing is converting raw text data into well-defined linguistically meaningful units and structures: words, clauses, sentences and documents. Because these units are passed to all further processing and analysis stages, the reduction of unnecessary noise, complexity and dimensionality in their digital representation is needed (Indurkhya & Damerau, 2010). To obtain the raw data, we converted the editorials, stored as PDF, into plain text files with UTF-8-character encoding.

Texts in Finnish and other languages contain instances of multiple word forms for a single base word. An *inflected* word expresses different grammatical categories such as tense and case. Inflection increases the dimensionality of text because a single base word can appear in numerous word forms within a text. Reducing these inflected word forms to their base form decreases the dimensionality. Two common examples of the reduction process are stemming, which maps words that refer to the same basic concept to a single root/stem (*family* and *families* become *famil*), and lemmatisation, which maps words to their *dictionary form* (Grimmer & Stewart, 2013). Finnish is an agglutinative and morphologically rich language. In theory, Finnish can have thousands of different inflected word forms where the original root is fused with prefixes, infixes and/or suffixes (Arppe, 2006). This means that the initial dimensionality of the texts is high (Nicolai & Kondrak, 2016). Koljonen et al. (2020, p. 5) did not use stemming in their application of Wordfish “given the evidence that stemming may combine words with different meanings”. Lemmatisation avoids this pitfall by reducing words to their base forms. In this study, we use the lemma as the unit of analysis. To obtain lemmas, we used the Turku Neural Parser Pipeline (TNPP), which is a highly accurate tool for converting Finnish, Swedish and English texts into a linguistically rich syntax standard called CoNLL-U (Kanerva et al., 2018). CoNLL-U format displays numerous linguistic features – part-of-speech tags, morphological features and dependency relations, for example – that TNPP deducts from the texts using a well-trained algorithm. In this study, we only use the lemma and part-of-speech tags when making decisions.

Words that carry a low semantic value, such as punctuation, interjections, symbols and pronouns, were automatically stripped from all the documents. Lemmas that consist of fewer than three characters were also removed. In addition to the standard removal of irrelevant words, the following threshold-based filtering conditions were used to strip more irrelevant words: the removal of words with a frequency of less than two; the combined removal of words with a frequency of less than three; and the removal of words unique to a single document within the corpus. The use of additional filtering increased the substantial validity of the analyses.

## Results and discussion

### Gambling policy positions of the editorials of *Helsingin Sanomat*

**Figure 1.**
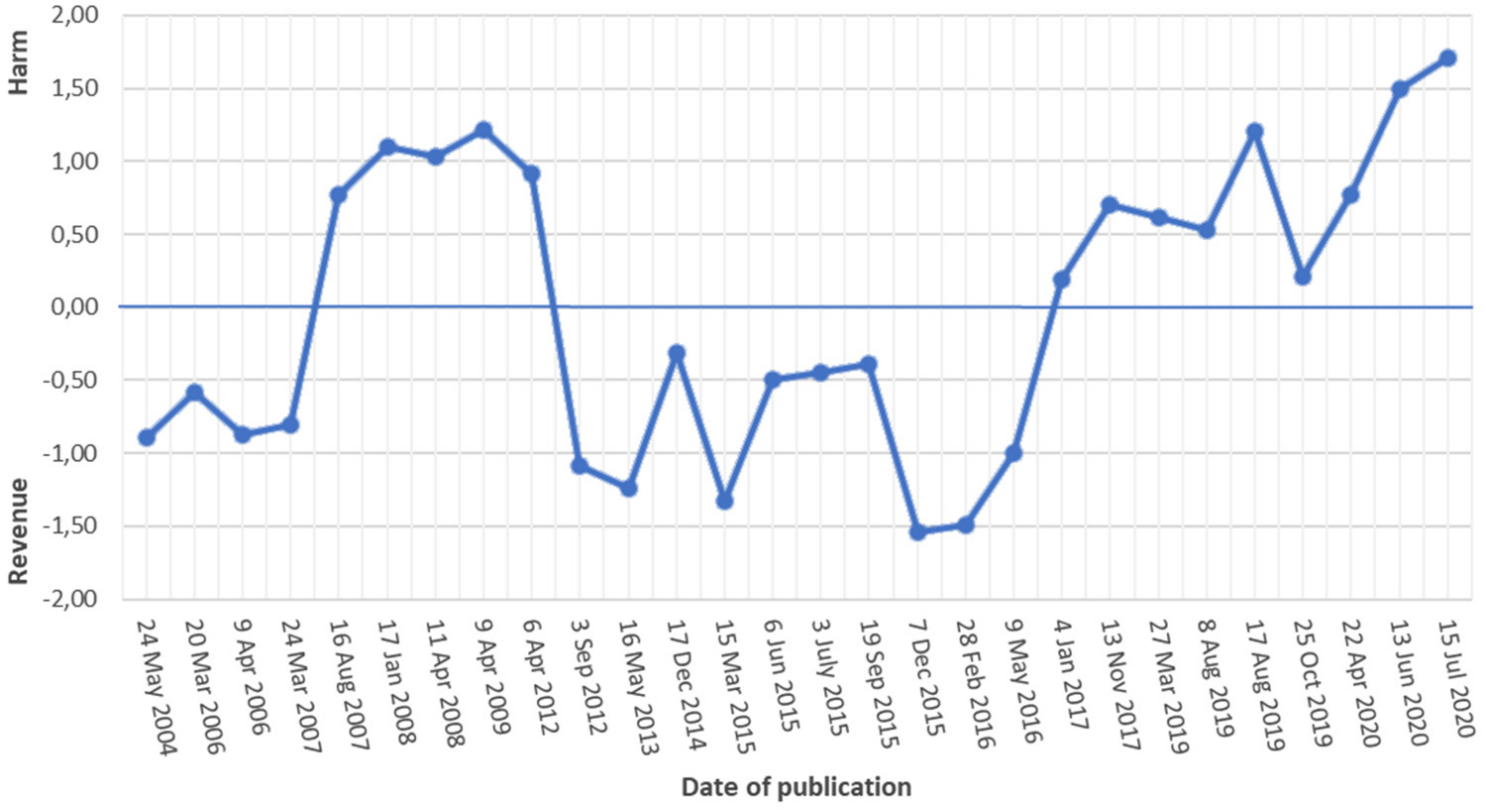
Gambling policy position estimates of the editorials of *Helsingin Sanomat* between 2004 and 2020.

The first analysis provides the longest time series on policy positions enabled by the data and is based on all the editorials published in *Helsingin Sanomat* between 2004 and 2020 (*N* = 28). In the raw data there were 7,763 words and 1,968 unique words. After the standard removal of irrelevant words, and removal of words with a frequency of less than two, there were 3,179 words and 700 unique words in the final analysis. In this analysis, position estimates were calculated for each editorial. The most negative values of the estimates are closest to the revenue end of the dimension and the most positive values of the estimates are closest to the harm end of the dimension. The results show (Figure 1) that despite a great deal of variance, there is a clear upward trend from the revenue end to the harm end of the dimension after 2016. The current monopoly operator Veikkaus started its operations in January 2017. It seems that the change in the policy position of *Helsingin Sanomat* corresponds to this. In addition, it is noteworthy that between 2007 and 2009 the position of *Helsingin Sanomat* was also closer to the harm end of the dimension. Considerable changes in the gambling policy positions of *Helsingin Sanomat* have thus taken place previously.

The position estimates for every *Helsingin Sanomat* editorial and the approximated confidence intervals are presented in Table 2. There are differences between the position estimates based on the actual data and the approximated or simulated estimates. This is likely to be an indication of the shortness of the editorials and the high number of words used infrequently (Proksch & Slapin, 2009). Also, the rather wide confidence intervals indicate the same.

**Table 2.** The policy position estimates of the editorials of *Helsingin Sanomat* and the simulated position estimates with 95% confidence intervals (CIs).

Editorial	Position estimate	Simulated position	95% CI
24 May 2004	−0.89^a^	−0.70	−0.87, −0.53
20 Mar 2006	−0.58	−0.50	−0.75, −0.22
9 Apr 2006	−0.87	−0.77	−1.01, −0.52
24 Mar 2007	−0.81	−0.72	−0.88, −0.53
16 Aug 2007	0.77	0.70	0.51, 0.89
17 Jan 2008	1.10	1.05	0.76, 1.30
11 Apr 2008	1.03	0.96	0.69, 1.23
9 Apr 2009	1.22	1.19	0.98, 1.39
6 Apr 2012	0.92	0.85	0.66, 1.04
3 Sep 2012	−1.09	−1.04	−1.19, −0.87
16 May 2013	−1.24	−1.23	−1.42, −1.03
17 Dec 2014	−0.31	−0.26	−0.54, 0.06
15 Mar 2015	−1.33	−1.35	−1.56, −1.15
6 Jun 2015	−0.50	−0.43	−0.69, −0.16
3 July 2015	−0.45	−0.38	−0.56, −0.20
19 Sep 2015	−0.39	−0.33	−0.55, −0.10
7 Dec 2015	−1.54	−1.71	−2.01, −1.46
28 Feb 2016	−1.49	−1.63	−1.94, −1.39
9 May 2016	−1.00	−0.93	−1.17, −0.65
4 Jan 2017	0.19	0.15	−0.04, 0.37
13 Nov 2017	0.70	0.62	0.30, 0.92
27 Mar 2019	0.62	0.54	0.25, 0.85
8 Aug 2019	0.53	0.46	0.20, 0.73
17 Aug 2019	1.21	1.18	0.94, 1.40
25 Oct 2019	0.21	0.18	−0.16, 0.49
22 Apr 2020	0.77	0.69	0.42, 0.95
13 Jun 2020	1.50	1.55	1.30, 1.77
15 Jul 2020	1.71	1.87	1.69, 2.08

^a^ The estimate is outside the simulated CIs, which indicates that there are possibly a notable number of words in the editorial in question that are infrequent in the other editorials.

As this was the analysis with the smallest subset of the raw data, and because we had no prior knowledge of the policy positions of the newspapers, an expert survey was conducted, and the estimates of the experts were compared to the Wordfish estimates (Supplementary Appendix C, online). The correlation between the Wordfish estimates and the expert survey was 0.75 (sig. 0.002). The high correlation between the estimates shows that despite the rather small number of words in the data, the validity of the Wordfish estimates is fairly good at the level of single editorials.

Substantively, the first analysis captured the revenue–harm policy dimension well (Table 3). Many of the words with the lowest negative word weights are related to the economic aspects of gambling and refer to the initial plans of establishing a new casino near the border between Finland and Russia and to the later decision of establishing the casino elsewhere in Finland. The majority of the words with the highest positive word weights refer to gambling harm and harm prevention. Words related to the one of the most harmful forms of gambling, electronic gambling machines (EGMs) (e.g., Yücel et al., 2018), and their placement are foregrounded.

**Table 3.** The top 15 words placing the editorials and newspapers on the revenue–harm dimension.

The editorials of *Helsingin Sanomat* between 2004 and 2020				*Aamulehti*, *Helsingin Sanomat*, *Kaleva* and *Turun Sanomat* between 2014 and 2020				Editorials of the major Finnish newspapers between pairs of years 2006–2007 and 2019–2020				
Harm	Word weight	Revenue	Word weight	Harm	Word weight	Revenue	Word weight	Harm	Word weight	Revenue	Word weight	
machine	6.035	Russian tourist	−5.701	machine	4.639	Russian tourist	−3.785	identification	4.602	charity		−2.612
identification	5.825	capital region	−5.701	to operate	4.272	capital region	−3.785	to decrease	3.714	to determine		−2.612
mandatory	5.825	permission	−5.135	to close	4.164	media	−2.736	regulation	3.714	maintenance		−2.612
gaming machine	5.622	municipality	−5.076	gambling outlet	4.114	government	−2.440	prevention	3.325	social work		−2.612
prevention	5.074	cherish	−5.076	distribution	3.838	to remember	−2.366	arcade	3.103	holder		−2.576
to start	5.074	rouble	−4.906	individual	3.211	casino	−2.309	critical	3.103	million		−2.381
gambling machine	4.938	be delayed	−4.906	to place in a decentralised way	3.211	cut	−2.228	reduction	2.920	gambling operator		−2.068
to open	4.417	withdraw	−4.906	require	3.211	move	−2.228	to hear	2.855	actively		−2.068
to close	4.417	to establish	−4.739	placing	3.174	economic policy	−2.214	quarter	2.855	to assure		−1.969
to place	4.106	pajazzo game	−4.422	to wait	3.048	a plan	−2.138	decreasing	2.855	a regulation		−1.969
online casino	3.825	establishment	−4.308	mandatory	2.989	ministry of interior	−2.089	gambling machine	2.739	to interpret		−1.969
pensioner	3.825	pajazzo	−4.093	to remove	2.985	government program	−2.089	gambling monopoly	2.578	gambling revenue		−1.969
closing	3.825	homogeneous culture	−4.093	to raise	2.931	to compete	−2.017	removal	2.575	child protection		−1.969
means	3.825	outside	−3.879	gambling addiction	2.882	ministerial committee	−1.999	Finnish Competition and Consumer Authority	2.575	interpretation		−1.969
gambling outlet	3.721	channel	−3.852	measure	2.872	to own	−1.701	family	2.575	disabled		−1.969

### Comparison of the gambling policy positions of *Aamulehti, Kaleva, Helsingin Sanomat* and *Turun Sanomat*

The second comparative analysis is based on the editorials published in *Aamulehti*, *Kaleva*, *Helsingin Sanomat* and *Turun Sanomat* between 2014 and 2020 (*N* = 36). The rationale behind this analysis is to see whether there are possible differences in the trajectories of changes in the gambling policy positions of the newspapers. In the raw data there were 11,062 words and 2,561 unique words, and after the standard removal of words and removal both of words with a frequency of less than three and of words unique to a single document within the corpus, there were 3,890 words and 522 unique words in the final analysis. The editorials published in each newspaper in the same year were pooled and treated as a single text in the analysis. The starting year for the analysis was 2014 because discussions on the merging of the existing three Finnish state-controlled gambling operators started then (see Selin et al., 2019). Moreover, for every year during this period there were editorials published in at least two different newspapers.

**Figure 2.**
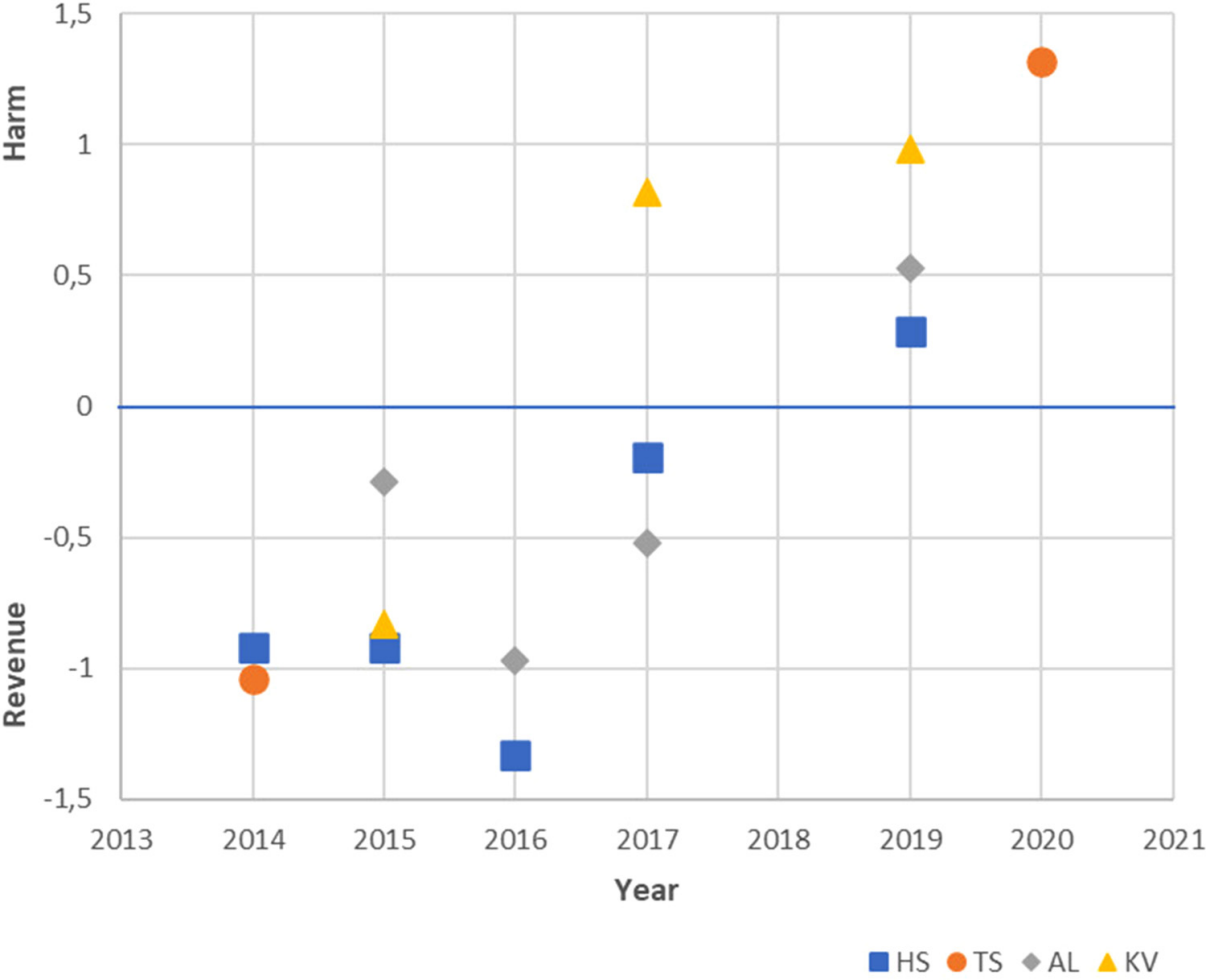
Gambling policy position estimates of *Kaleva* (*KV*), *Aamulehti* (*AL*), *Turun Sanomat* (*TS*) and *Helsingin Sanomat* (*HS*) between 2014 and 2020.

The results of the second analysis show that the positions of all four newspapers have moved clearly towards the harm end of the policy dimension over the latter years of the observation period (Figure 2). There are, however, some differences between the newspapers: the position of *Kaleva* was already leaning towards the harm end in 2017, whereas *Helsingin Sanomat* and *Aamulehti* were at the same time inclined towards the revenue end of the dimension. Confidence intervals were quite wide, which indicates that the stability of the results could be better (Table 4).

**Table 4.** The policy position estimates of *Aamulehti* (*AL*), *Helsingin Sanomat* (*HS*), *Kaleva* (*KV*), and *Turun Sanomat* (*TS*) Between 2014 and 2020 and the simulated policy position estimates with 95% confidence intervals (CIs).

Year	Position estimate	Simulated position	95% CI
*HS* 2014	−0.92	−0.90	−1.07, −0.73
*TS* 2014	−1.04	−1.03	−1.23, −0.81
*AL* 2015	−0.29	−0.28	−0.55, 0.02
*HS* 2015	−0.92	−0.91	−1.02, −0.81
*KV* 2015	−0.83	−0.81	−1.01, −0.60
*AL* 2016	−0.97	−0.96	−1.13, −0.79
*HS* 2016	−1.33	−1.38	−1.57, −1.21
*AL* 2017	−0.52	−0.49	−0.66, −0.29
*HS* 2017	−0.19	−0.19	−0.38, 0.07
*KV* 2017	0.82	0.79	0.48, 1.07
*AL* 2019	0.53	0.52	0.24, 0.78
*HS* 2019	0.29	0.28	0.08, 0.51
*KV* 2019	0.98	0.97	0.80, 1.12
*TS* 2019	1.54	1.54	1.37, 1.71
*HS* 2020	1.52	1.52	1.34, 1.70
*TS* 2020	1.32	1.32	1.04, 1.57

Substantively, the results seem valid (Table 3). The words related to the economic aspects of gambling mainly refer to either the establishment of the second casino or the discussions on merging the three state-controlled gambling operators (Selin et al., 2019). The words with the highest positive weights refer to harms related to EGMs and their placement.

### The gambling policy positions of the major newspapers

In the third analysis, all the editorials published in all the newspapers over two consecutive years were pooled (*N* = 56). The purpose of this analysis was to be able to observe the changes in the policy positions on a more general level. In the raw data there were 14,978 words and 3,261 unique words, and after the standard removal of words and removal of words unique to a single document, there were 5,849 words and 912 unique words in the final analysis. To avoid over-representation, it was decided that in every pool there needed to be editorials from at least two different newspapers. The editorials published during the paired years were then treated as single texts in the analysis.

**Figure 3.**
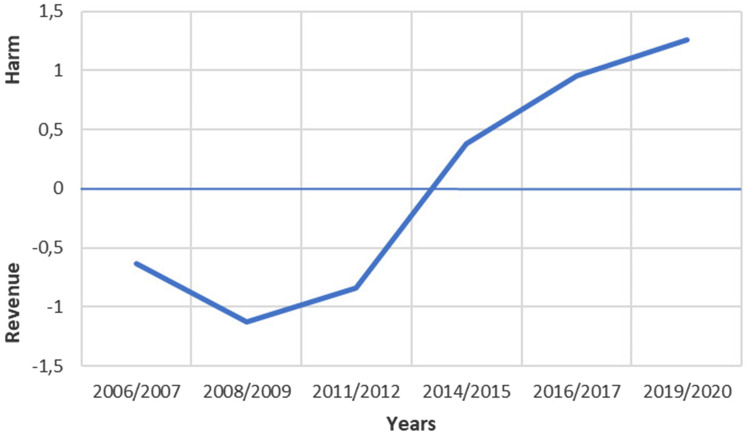
The gambling policy positions of major Finnish newspapers between 2006/2007 and 2019/2020.

The results of the third analysis largely corroborate the results of the previous two analyses: as time goes by there is a discernible shift in the policy positions of the newspapers towards the harm end of the policy dimension (Figure 3). However, the results of the third analysis indicate that this change gradually started to take place well before the wave of criticism towards the monopoly operator Veikkaus and the regulatory framework in 2019, and even before the merging of the three gambling operators into one in 2017. The confidence intervals are not as wide as in the two previous analyses, which indicates that combining the texts into larger wholes increased the accuracy of the estimates (Table 5).

**Table 5.** The gambling policy position estimates of the major Finnish newspapers and the simulated position estimates with 95% confidence intervals (CIs).

Years	Position estimate	Simulated position	95% CI
2006/2007	−0.63	−0.62	−0.71, −0.53
2008/2009	−1.13	−1.12	−1.21, −1.03
2011/2012	−0.84	−0.81	−0.91, −0.72
2014/2015	0.38	0.31	0.20, 0.43
2016/2017	0.95	0.93	0.83, 1.03
2019/2020	1.26	1.31	1.23, 1.39

Substantively, the results are valid. The words with the most negative weights are related to the use of gambling revenue for different societal purposes such as child protection, social work or substance abuse treatment and prevention (Table 3). The words with the highest positive weights still refer mostly to EGMs.

## Conclusions

The results of all the analyses support our hypothesis, according to which the policy positions of the newspapers would have moved towards the harm end of the policy dimension between 2004 and 2020. The overall change in the policy positions started possibly before the new monopoly operator Veikkaus started its operations in 2017. However, the shift in policy positions has been the most evident over the latter years of the period, which could be the result of the public criticism of the regulatory framework and operations of the gambling monopoly after 2019. It is possible that the overall shift in the gambling policy positions of the newspapers, growing media interest in gambling and the wave of criticism towards the gambling monopoly and gambling policy in Finland are manifestations of other larger changes in the societal and cultural position of gambling in Finland. The developments in Finland are similar to the counter-reactions that are a result of experiences of gambling harm, the increased availability and visibility of gambling, and that have led to policy reforms in many European countries, such as Italy, Spain and the United Kingdom (*Casino News Daily*, 2020; Rolando & Scavarda, 2018).

The results also show that the policy positions of the major Finnish newspapers follow different trajectories. These may be related to differences in the overall policies of the newspapers. Moreover, as the results of the analysis of the individual editorials of *Helsingin Sanomat* indicate, there can be considerable differences in policy positions when individual editorials published temporally close to each other are considered. One explanation for this is that newspaper editorials are very much tied to the content of topical gambling policy discussions. For example, the merger of the three Finnish state-controlled gambling operators was discussed a great deal between 2014 and 2017, but the discussion was more related to questions concerning the necessity of the merger and the benefits and justifications of the merger than gambling harm (Selin et al., 2019). In contrast, when the introduction of the 18-year age limit was under discussion between 2007 and 2009, the policy position in the editorials of *Helsingin Sanomat* were clearly at the harm end of the policy dimension. It is therefore likely that, at least on the level of individual editorials, the policy positions manifested in the editorials are reflections of the topical issues on the gambling policy agenda but not necessarily serious attempts to influence the political or public agenda. However, the overall changes in the gambling policy positions of the newspapers, especially over the last few years, are so noteworthy that it is unlikely that they are mere reactions to topical issues.

Studying the policy positions of different stakeholders with the help of automated content analysis methods such as Wordfish offers great opportunities for gambling policy research insofar as there are large enough textual data sets available and other relevant pre-conditions for the chosen method can be fulfilled. Automated content analysis methods would make it possible to estimate the impact of gambling policy positions of different stakeholders on the public and political agenda as well as on official gambling policy. Supervised automated content analysis has already been applied to the study of the impacts of lobbying on different policies with compelling results (Costa et al., 2014; Klüver, 2009). Moreover, a comparison of public health discourse with the responsible gambling discourse favoured by the gambling industry could be an interesting new application for Wordfish. Studying the degree to which official gambling policies with the aim of preventing gambling harm are in line with the best research evidence could be another possible application of the supervised automated content analysis method.

This study has several limitations. First, there is over-representation of the editorials of the biggest daily newspaper, *Helsingin Sanomat*, in the raw data. Second, the overall size of the raw data was quite small. With a larger data set the confidence intervals would have been narrower, and it is likely that the stability of the word weight estimates would have increased (Proksch & Slapin, 2009; Slapin & Proksch, 2008). Third, as this a pioneering study, checking the correspondence of all the results with gambling policy estimates from other methods was not possible.

Future research will show whether the clear overall shift in the gambling policy positions of the major Finnish newspapers will have influence on the political agenda and gambling policy (Van Aelst & Walgrave, 2011). The results nevertheless indicate that this is possible: the change in the gambling policy positions of the newspapers could result in more news coverage on gambling harm and on gambling policy, and this could in turn contribute to increased public awareness of gambling harm. In addition, there are active researchers and citizens in Finland who endeavour to keep gambling harm and gambling policy on the public agenda. Substantial policy change as a result of media reporting is usually a rare and slow process, but insofar as political actors consider the attention of media to be a reflection of public opinion, the chances of policy change are higher (Walgrave & Van Aelst, 2006). Due to the interplay between the media, politics, and the public, it is likely that the importance of prevention and reduction of gambling harm will be recognised and addressed when gambling policy is formulated in Finland in the near future. An example of prevention would be a significant reduction in the current high availability of EGMs (Raisamo et al., 2019). This would help to reduce the tension between gambling harm and gambling revenue that currently characterises gambling policy in Finland. Generally speaking, if the gambling policy positions of media and other stakeholders change, this can facilitate promoting harm prevention policies.

## Supplemental Material

sj-docx-1-nad-10.1177_14550725221083438 - Supplemental material for Gambling policy positions of Finnish newspapers between 2004 and 2020: An automated content analysis Click here for additional data file. Supplemental material, sj-docx-1-nad-10.1177_14550725221083438 for Gambling policy positions of Finnish newspapers between 2004 and 2020: An automated content analysis by Jani Selin and Riku Nyrhinen in Nordic Studies on Alcohol and Drugs

sj-docx-2-nad-10.1177_14550725221083438 - Supplemental material for Gambling policy positions of Finnish newspapers between 2004 and 2020: An automated content analysis Click here for additional data file. Supplemental material, sj-docx-2-nad-10.1177_14550725221083438 for Gambling policy positions of Finnish newspapers between 2004 and 2020: An automated content analysis by Jani Selin and Riku Nyrhinen in Nordic Studies on Alcohol and Drugs

sj-docx-3-nad-10.1177_14550725221083438 - Supplemental material for Gambling policy positions of Finnish newspapers between 2004 and 2020: An automated content analysis Click here for additional data file. Supplemental material, sj-docx-3-nad-10.1177_14550725221083438 for Gambling policy positions of Finnish newspapers between 2004 and 2020: An automated content analysis by Jani Selin and Riku Nyrhinen in Nordic Studies on Alcohol and Drugs

## Notes

1. The autonomous Åland Islands form a separate jurisdiction in gambling policy and are not considered in this study.

2. The discussions resulted in the introduction of 18-year age limit for all gambling by 2011 (for example Selin, 2019).

3. For example, a *Turun Sanomat* editorial was excluded because it discussed the decision of the Finnish Communications Regulatory Authority to end the broadcasting of Lotto draws on the channels of the national broadcasting company YLE. The authority considered the broadcasting to be against the Act on YLE.
