# Supplementary material for: Gambling policy positions of Finnish newspapers between 2004 and 2020: An automated content analysis
Source: Nordisk Alkohol Nark. 2022 Aug 11;39(6):605–22. doi: 10.1177/14550725221083438 (PMC9703367; doi:10.1177/14550725221083438)
Supplement: sj-docx-1-nad-10.1177_14550725221083438 - Supplemental material for Gambling policy positions of Finnish newspapers between 2004 and 2020: An automated content analysis [file sj-docx-1-nad-10.1177_14550725221083438.docx]

**Appendix A**

The newspaper editorials in the raw data

*Aamulehti*

The Editorial Board. (2015, December 14). Uhkapelit ulos kaupoista [Remove gambling from shops]. *Aamulehti*. <https://www.aamulehti.fi/>

The Editorial Board. (2016, January 21). Kasino Tampereelle [Casino to Tampere]. *Aamulehti*. <https://www.aamulehti.fi/>

The Editorial Board. (2016, May 5). Tampereen on tehtävä uhkapelistä mahdollisuus [Tampere needs to make an opportunity out of gambling]. *Aamulehti*. <https://www.aamulehti.fi/>

The Editorial Board. (2017, January 8). Uhkapeli ja pelin uhka [Gambling game and game as a gamble].

*Aamulehti*. <https://www.aamulehti.fi/>

The Editorial Board. *Aamulehti*. (2017, November 25). Uhkapelit pois baareista – rahapeleille oikea paikka ovat valvotut kasinot ja pelisalit [Remove gambling from bars: The right places for gambling are casinos and arcades]. <https://www.aamulehti.fi/>

The Editorial Board. (2018, September 4). Kilpailuvirastolta tärkeä kannanotto Veikkauksen monopoliaseman ongelmista [The Finnish Competition and Consumer Authority makes an important statement regarding the problems of the monopoly status of Veikkaus]. *Aamulehti*. <https://www.aamulehti.fi/>

The Editorial Board. (2019, January 27). Kasinokaupunki Tampereen on oltava tarkkana: peli pyörii, raha puhuu, unohtuuko ihminen? [Casino city Tampere has to be alert: The game is on, money talks, are the people forgotten? *Aamulehti*. <https://www.aamulehti.fi/>

The Editorial Board. (2019, March 31). Rahapelien haittoja pitää pystyä vähentämään - Peliautomaatit pois ruokakaupoista? [There has to be the ability to reduce gambling harm: Removal of electronic gambling machines from grocery stores?]. *Aamulehti*. <https://www.aamulehti.fi/>

*Helsingin Sanomat*

The Editorial Board. (2004, May 24). Internet uhkaa rahapelien monopoleja [Internet threatens gambling monopolies]. *Helsingin Sanomat*. <https://www.hs.fi>

The Editorial Board. (2006, March 20). Pelimonopoliin oltava oikeus [Gambling monopoly needs to be entitled]. *Helsingin Sanomat*. <https://www.hs.fi>

The Editorial Board. (2006, April 4). Vedonlyöntimonopolia puolustettava [Monopoly on sports betting needs to be defended]. *Helsingin Sanomat*. <https://www.hs.fi>

The Editorial Board. (2008, January 17). Rahapelien haittoja voidaan ehkäistä [Gambling harm can be prevented]. *Helsingin Sanomat*. <https://www.hs.fi>

The Editorial Board. (2008, April 11). Esitys arpajaislaiksi vaatii lisätyötä [Proposal for Lotteries Act needs extra work]. *Helsingin Sanomat*. <https://www.hs.fi>

The Editorial Board. (2009, April 9). Osa sosiaalityöstä on yhä rahapelituottojen varassa [Part of social work funding still dependent on gambling revenue]. *Helsingin Sanomat*. <https://www.hs.fi>

The Editorial Board. (2012, April 6). Pelirahan uusi jako vaarantaa järjestötyötä [The new distribution of gambling revenue endangers work done in non-governmental organisations].  *Helsingin Sanomat*. <https://www.hs.fi>

The Editorial Board. (2012, September 3). Pelihimo vaivaa päättäjiä [Decision-makers suffer from gambling addiction]. *Helsingin Sanomat*. <https://www.hs.fi>

The Editorial Board. (2013, May 16). Rahapeli ratkaisee [Gambling rules]. *Helsingin Sanomat*. <https://www.hs.fi>

The Editorial Board. (2014, December 17). Hallitus ajaa peliremonttia [Government pushes for gambling reform]. *Helsingin Sanomat*. <https://www.hs.fi>

The Editorial Board. (2015, March 15). Yhtenäiskulttuuri murenee [Cultural homogeneity is crumbling]. *Helsingin Sanomat*. <https://www.hs.fi>

The Editorial Board. (2015, June 6). Loton alamäki hermostuttaa [The decline of Lotto is upsetting]. *Helsingin Sanomat*. <https://www.hs.fi>

The Editorial Board. *Helsingin Sanomat*. (2015, July 3). Rahapeliyhtiöt on järkevää yhdistää [Merging gambling operators is reasonable]. <https://www.hs.fi>

The Editorial Board. (2015, September 19). Veikkauksen, RAY:n ja Fintoton yhdistäminen on järkevää [Merging Veikkaus, RAY and Fintoto is reasonable]. *Helsingin Sanomat*. <https://www.hs.fi>

The Editorial Board. (2015, December 7). Minne tulisi toinen kasino? [Where to place the second casino?]. *Helsingin Sanomat*. <https://www.hs.fi>

The Editorial Board. (2016, February 28). Kaikki haluavat kasinon [Everybody wants to have a casino]. *Helsingin Sanomat*. <https://www.hs.fi>

The Editorial Board. (2016, May 9). Kaksi tapaa puhua peleistä [Two ways of talking about gambling]. *Helsingin Sanomat*. <https://www.hs.fi>

The Editorial Board. (2017, January 4). Pelaaminen saa uuden valvojan [New overseer for gambling]. *Helsingin Sanomat*. <https://www.hs.fi>

The Editorial Board. (2017, November 13). Rahapeliriippuvuus peittyy monen kauniin asian alle [Many beautiful things conceal gambling addiction]. *Helsingin Sanomat*. <https://www.hs.fi>

The Editorial Board. *Helsingin Sanomat*. (2019, March 27). Rahapelit ovat edelleen tulonsiirto köyhiltä ja peliongelmaisilta [Gambling is still income transfer from the poor and problem gamblers]. <https://www.hs.fi>

The Editorial Board. (2019, August 8). Veikkausta pitäisi katsoa nykyistä useammin kriittisellä silmällä [Veikkaus should be observed more often with a critical eye]. *Helsingin Sanomat*. <https://www.hs.fi>

The Editorial Board. (2019, August 17). Veikkauksen on vihdoin otettava rahapelien haitat tosissaan [Veikkaus needs to finally take gambling harm seriously]. *Helsingin Sanomat*. <https://www.hs.fi>

The Editorial Board. (2019, October 25). Veikkauksen hallinto ja valvonta pitäisi erottaa tuottojen jakamisesta [The governance and supervision of Veikkaus should be separated from the distribution of funds]. *Helsingin Sanomat*. <https://www.hs.fi>

The Editorial Board. (2020, April 22). Yhteisten rahojen jakaminen kaipaa myös normaaliaikoina avoimuutta [Allocation of public funds also needs to be transparent in normal times]. *Helsingin Sanomat*. <https://www.hs.fi>

The Editorial Board. (2020, June 13). Poikkeuskevät antaa eväät arvioida rahapeliautomaattien vaikutuksia [Exceptional spring gives opportunity for assessing the impacts of electronic gambling machines]. *Helsingin Sanomat*. <https://www.hs.fi>

The Editorial Board. (2020, July 15). Pelihaittojen vähentämisen vuoksi pakollinen tunnistautuminen peliautomaateille on tärkeää [In order to reduce gambling harm, mandatory player identification for electronic gambling machines is important]. *Helsingin Sanomat*. <https://www.hs.fi>

*Kaleva*

The Editorial Board. (2015, July 8). Rahapelit yhteen yhtiöön [Gambling into a single company]. *Kaleva*. <https://www.kaleva.fi/>

The Editorial Board. (2017, November 20). Ei pelkkää lottovoittoa (Not just lottery winnings]. *Kaleva*. <https://www.kaleva.fi/>

The Editorial Board. *Kaleva*. (2019, March 28). Huomio haittojen ehkäisyyn – rahapelaamisella on myös kolikon toinen puoli [Attention to harm prevention: There is also the other side of the coin in gambling]. <https://www.kaleva.fi/>

The Editorial Board. (2019, August 14). Miten Veikkauksen monopolin voisi purkaa – kävisikö Ruotsin lisenssimalli? [How could the monopoly of Veikkaus be terminated: Would the licence model of Sweden suit? *Kaleva*. <https://www.kaleva.fi/>

The Editorial Board. *Kaleva*. (2019, September 6). Veikkaukselle syystäkin kireämmät suitset – hyvä tarkoituskaan ei pyhitä kyseenalaisia rahanhankintakeinoja [Veikkaus needs a tighter bridle for a reason: Good ends do not justify the questionable means of making money]. <https://www.kaleva.fi/>

The Editorial Board. (2019, October 28). Veikkauksen maine liipaisimella – peliyhtiön valvonta kaipaa selkeyttä ja vastuullisuutta [The reputation of Veikkaus is on a razor’s edge: The supervision of the gambling company requires clarity and responsibility]. *Kaleva*. <https://www.kaleva.fi/>

The Editorial Board. (2019, November 6). Veikkaus tarkkailuluokalle – ryhtiliikkeeltä on lupa odottaa pelihaittojen vähenemistä [Veikkaus on a trial period: Improvement is expected to result in reduction of gambling harm]. *Kaleva*. <https://www.kaleva.fi/>

*Keskisuomalainen*

The Editorial Board. (2019, July 27). Veikkauksen markkinointiviestiä on syytä rajoittaa [There is a need to limit marketing communications of Veikkaus]. *Keskisuomlainen*. <https://www.ksml.fi/>

The Editorial Board. (2019, August 28). Veikkauksessa on poliittisen piilokorruption piirteitä [There are signs of hidden political corruption in Veikkaus]. *Keskisuomlainen*. <https://www.ksml.fi/>

The Editorial Board. (2019, Septembet 6). Veikkauksen ”sivupersoonalle” halutaan rajat [Limits wanted for the “alter ego” of Veikkaus]. *Keskisuomlainen*. <https://www.ksml.fi/>

*Turun Sanomat*

The Editorial Board. (2007, July 31). Peliriippuvuus kasvaa [Gambling addiction increases]. *Turun Sanomat*. <https://www.ts.fi/>

The Editorial Board. (2007, November 20). Veikkausvarat jaettava oikein [Gambling revenue needs to be allocated correctly]. *Turun Sanomat*. <https://www.ts.fi/>

The Editorial Board. (2008, January 18). Pelivalvonta voi johtaa harhaan [Supervision of gambling can lead astray]. *Turun Sanomat*. <https://www.ts.fi/>

The Editorial Board. (2008, February 8). Pelilantteja uhkaa sukupuutto [Gambling coins in danger of extinction]. *Turun Sanomat*. <https://www.ts.fi/>

The Editorial Board. (2008, April 20). Pelirahaa karkaa ulkomaille [Gambling money goes to offshore].

*Turun Sanomat*. <https://www.ts.fi/>

The Editorial Board. (2011, March 11). Vanhempien vastuu korostuu [The responsibility of parents will be emphasised]. *Turun Sanomat*. <https://www.ts.fi/>

The Editorial Board. (2011, June 6). Monopolit avoimuuden kontrolliin [Monopolies under the control of transparency]. *Turun Sanomat*. <https://www.ts.fi/>

The Editorial Board. (2011, October 10). Rahanjako lailliselle pohjalle [Allocation of money to legitimate basis]. *Turun Sanomat*. <https://www.ts.fi/>

The Editorial Board. (2014, December 8). Rahapelien kanssa ei pitäisi hötkyillä [No need to rush with gambling]. *Turun Sanomat*. <https://www.ts.fi/>

The Editorial Board. (2019, September 7). Veikkauksen jakovara vähenee [Veikkaus assets decrease]. *Turun Sanomat*. <https://www.ts.fi/>

The Editorial Board. (2019, November 1). Veikkaus pesee käsiään [Veikkaus washes its hands]. *Turun Sanomat*. <https://www.ts.fi/>

The Editorial Board. (2019, December 28). Peliriippuvuutta ehkäistävä ennalta [Gambling addiction needs to be prevented]. *Turun Sanomat*. <https://www.ts.fi/>

The Editorial Board. (2020, July 15). Veikkauksella ristiriitaisia tavoitteita [Veikkaus has contradictory objectives]. *Turun Sanomat*. <https://www.ts.fi/>
