# Supplementary material for: Gambling policy positions of Finnish newspapers between 2004 and 2020: An automated content analysis
Source: Nordisk Alkohol Nark. 2022 Aug 11;39(6):605–22. doi: 10.1177/14550725221083438 (PMC9703367; doi:10.1177/14550725221083438)
Supplement: sj-docx-2-nad-10.1177_14550725221083438 - Supplemental material for Gambling policy positions of Finnish newspapers between 2004 and 2020: An automated content analysis [file sj-docx-2-nad-10.1177_14550725221083438.docx]

**Appendix B**

Five Steps of the Expectation Maximisation in Wordfish

First, starting values are obtained for word- and actor-fixed effects (ψ and α, respectively) by calculating the following from a (n, m) word document matrix, where the cell, w_ij_, represents the frequency of word *j* in document *i*:

$$\alpha_{i}=\log\left( \frac{\sum_{j=1}^{m} w_{ij}*\frac{1}{m}}{\sum_{j=1}^{m} w_{1j}*\frac{1}{m}} \right), \forall i\in\left\{ 1,\ldots,n \right\}$$

$$\psi_{j}=\log\left( \sum_{i=1}^{n} w_{ij}*\frac{1}{m} \right), \forall j\in\left\{ 1,\ldots,m \right\}$$

The starting values for the word- and actor-fixed effects are subtracted from the logged word frequencies to create a matrix from which we then extract – through singular value decomposition – the left- and right-singular vectors as starting values for ω and β, respectively.

Second, the actor parameters, α and ω, are estimated as conditional on the expectations for word parameters. During the first iteration, the expectation of word parameters equals their starting values calculated in the first step. In the following iterations, the word values of the previous iteration will be used. To identify the model, the first actor-fixed effect, α, is set to zero for calculating the estimate for the first actor position parameter, ω. The following log-likelihood is maximised for each actor *i*:

$$\sum_{j=1}^{m} \left( -\lambda_{ij}+\ln\left( \lambda_{ij} \right)*y_{ij} \right)$$

where

$$\lambda_{ijt}=\exp\left( \alpha_{i}+\psi_{j}^{start}+\beta_{j}^{start}*\omega_{i} \right)$$

Third, the process from the second step is repeated for word parameters, *β* and *ψ*, with the expectation of actor parameters, used as starting values, equalling the values calculated in the second step. For each word *j*, we maximise the log-likelihood:

$$\sum_{i=1}^{n} \left( -\lambda_{ij}+\ln\left( \lambda_{ij} \right)*y_{ij} \right)-\left( \frac{\beta_{j}^{2}}{2}*\sigma^{2} \right)$$

where

$$\lambda_{ij}=\exp\left( \alpha_{i}^{step2}+\psi_{j}+\beta_{j}*\omega_{i}^{step2} \right)$$

The prior belief that *β*s are distributed normally with a mean of zero and a standard deviation of *σ* is included in this log-likelihood. This prevents infrequent words from carrying infinite weight.

Fourth, the log-likelihood of the model is defined as the sum of the individual word log-likelihoods from the third step, which are themselves calculated as conditional upon the actor log-likelihoods from step 2:

$$\sum_{j=1}^{m} \sum_{i=1}^{n} \left( -\lambda_{ij}+\ln\left( \lambda_{ij} \right)*y_{ij} \right)$$

Fifth, based on new expectations, actor and word parameters are re-estimated. This process is repeated until an acceptable level of convergence is reached. For the purposes of Wordfish, the convergence is measured as the sum of the differences of individual log-likelihoods from the fourth step (relative to the total log-likelihood) between successive iterations. In this study, the default threshold for the sum of differences relative to the total log-likelihood is 10^-7^, which is also the default value in the R implementation of Wordfish version 1.3 (Slapin & Proksch, 2008).
