# Supplementary material for: Gambling policy positions of Finnish newspapers between 2004 and 2020: An automated content analysis
Source: Nordisk Alkohol Nark. 2022 Aug 11;39(6):605–22. doi: 10.1177/14550725221083438 (PMC9703367; doi:10.1177/14550725221083438)
Supplement: sj-docx-3-nad-10.1177_14550725221083438 - Supplemental material for Gambling policy positions of Finnish newspapers between 2004 and 2020: An automated content analysis [file sj-docx-3-nad-10.1177_14550725221083438.docx]

**Appendix C**

Gambling Policy Position Estimates of the Experts on Selected Editorials of Helsingin Sanomat, and Corresponding Wordfish Estimates.

| Editorial | N | Min. | Max. | Experts | Std. Deviation | Wordfish |
| --- | --- | --- | --- | --- | --- | --- |
| 17 Dec 2014 | 10 | 1 | 2 | 1.30 | 0.48 | -0.31 |
| 15 Mar 2015 | 9 | 1 | 2 | 1.67 | 0.50 | -1.33 |
| 6 Jun 2015 | 8 | 2 | 5 | 3.13 | 0.84 | -0.50 |
| 3 Jul 2015 | 8 | 1 | 2 | 1.25 | 0.46 | -0.45 |
| 19 Sep 2015 | 9 | 1 | 2 | 1.22 | 0.44 | -0.39 |
| 7 Dec 2015 | 8 | 1 | 1 | 1.00 | 0.000 | -1.54 |
| 27 Mar 2019 | 10 | 2 | 4 | 3.20 | 0.63 | 0.62 |
| 8 Aug 2019 | 10 | 2 | 5 | 3.10 | 0.99 | 0.53 |
| 17 Aug 2019 | 8 | 4 | 5 | 4.38 | 0.52 | 1.21 |
| 25 Oct 2019 | 8 | 1 | 4 | 2.63 | 0.92 | 0.21 |
| 22 Apr 2020 | 10 | 1 | 4 | 2.00 | 0.82 | 0.77 |
| 13 Jun 2020 | 8 | 3 | 5 | 3.88 | 0.84 | 1.50 |
| 15 Jul 2020 | 8 | 2 | 5 | 4.00 | 0.93 | 1.71 |

*Note*. The response options in the expert survey were: 1) the text discusses gambling primarily as economic activity, 2) the text discusses gambling as economic activity, but other viewpoints are mentioned, 3) the text discusses gambling both as economic and harm-inducing activity, 4) the text discusses gambling as harm-inducing activity, but other viewpoints are mentioned, 5) the text discusses gambling primarily as harm-inducing activity, 6) no opinion.
